# Cultural factors in depressive experience and its severity: A case report

**DOI:** 10.1192/j.eurpsy.2023.1731

**Published:** 2023-07-19

**Authors:** C. Regueiro Martín-Albo, F. Mayor Sanabria, E. Expósito Durán, M. Fernández Fariña, M. Navas Tejedor

**Affiliations:** Hospital Clínico San Carlos, Madrid, Spain

## Abstract

**Introduction:**

For a very long time, anthropologists and psychiatrists have studied how the symptomatology of mental diseases varies among cultures. Different social environments approach depression in different ways, and cultural practices and meanings influence how it develops. Culture also affects how symptoms are felt and described, how treatments are chosen, how patients and doctors interact, how likely it is that certain events, like suicide, will occur, and how professionals behave. As a result, all of these circumstances must be taken into consideration when approaching the diagnosis and management of depressive disorders. To illustrate the above, we present the case of a 31-year-old man, originally from Nigeria, who was admitted to the hospital after a suicide attempt by precipitation onto the subway tracks.

**Objectives:**

(1) To describe the clinical particularities of this case, focusing on the diagnostic difficulties we faced derived from intercultural contrasts (2) To review cross-cultural differences in the symptomatology and its implication on severity of depressive disorders.

**Methods:**

A review of the patient’s clinical history and complementary tests performed was carried out. Likewise, a bibliographic review of the available scientific literature was also performed in relation to transcultural depressive experiences and its severity.

**Results:**

There is little evidence in favor of a direct link between sociocultural factors and severe depression, but we reviewed the arguments that look significant for further research. Depressive illnesses are found in all societies and their symptomatic expression varies culturally, particularly in terms of somatization and delusional ideas. Similarly, the social and individual representations of the disease depend on the culture, and some conceptual models can increase the effects of stigmatization. These cross-cultural variations could influence the care-seeking process and therefore modulate the evolution of the disease in the sense of greater severity.

**Conclusions:**

All societies experience depressive disorders, which exhibit symptoms that vary culturally, especially in terms of somatization and delusional beliefs.

The care-seeking process is affected by cross-cultural differences, and as a result, the disease’s progression may also be modulated in terms of increased severity.

When we ignore cultural factors in understanding, assessing, and treating depression, we are contributing to misdiagnosis and errors in patient management.

**Disclosure of Interest:**

None Declared

